# Understanding Child Abuse and Neglect Indications: Insights From Medical Students

**DOI:** 10.7759/cureus.62860

**Published:** 2024-06-21

**Authors:** Reem Alyoubi

**Affiliations:** 1 Department of Pediatrics, Faculty of Medicine, King Abdulaziz University, Jeddah, SAU

**Keywords:** child abuse, primary health care, childhood maltreatment, covid-19, can

## Abstract

Background: Child abuse is a severe issue that impacts medical professionals and patients globally. It can lead to discomfort, risk, or even the demise of a child. One of the most significant facets of a doctor’s work involves providing aid to those in need; since children are more dependent on others for care and safety, they should receive attention that is even more special in quality. Hence, this study aims to assess the views, professional experiences, and comprehension of Saudi Arabian medical students regarding child abuse and neglect (CAN).

Methodology: This study was carried out at Dr. Soliman Fakeeh Hospital. It involved 65 students who were either victims of child abuse or the relatives/family members of child abuse victims.

Results: The participants’ awareness of several critical components of CAN was considerable. However, their understanding regarding the reporting of CAN cases was rather meager. Furthermore, their views of and professional experience with CAN were not excellent in terms of quality.

Conclusions: Saudi Arabia should implement an extensive plan to prevent the abuse of children at any level, given the nation’s clinical experience and expertise in this field. The nation’s pediatricians should direct as well as assist in this process, thereby playing a major role in preventing and addressing CAN cases.

## Introduction

For young individuals, child abuse is a major source of stress that often has lasting consequences. Numerous disorders related to physical and mental health are associated with child abuse: post-traumatic stress disorder (PTSD), self-harming behavior, sexually transmitted infections, personality pathology, and common mental illnesses [1]. A variety of abusive acts that result in a child’s illness or death are considered forms of child maltreatment [2].

Throughout history, children have been killed, hurt, left hungry, abandoned, and neglected. Nevertheless, over the past 20 years, the problem of child abuse has received widespread medical attention. Child abuse is defined by the World Health Organization (WHO) as any form of physical and/or emotional ill-treatment, sexual abuse, neglect or careless treatment, or commercial or other exploitation that harms or threatens a child’s health, survival, development, or dignity in the context of an authority, trust, or responsibility relationship [3]. However, there exists a paucity of research or discussion on child abuse in the Arab world. This is alarming considering that over the period lasting from June 1997 to June 1998, the King Khalid University Hospital in Saudi Arabia received allegations of 13 incidences of child abuse and negligence [4].

Interestingly, medical professionals in Saudi Arabia kept records of child abuse and neglect private until the 1990s. Owing to a variety of factors, including a lack of training, the difficulty of reaching a conclusive diagnosis, the possibility of stigmatizing families, risks to their safety and well-being, and potential effects on their careers, some doctors in Saudi Arabia are still hesitant to diagnose child abuse or neglect. Moreover, some professionals find it difficult to follow social or legal requirements concerning cases of child abuse [5].

Notably, Al-Mahroos used the revision of medical reports issued between June 1987 and May 2005 to conduct research aimed at providing a general overview of the trends regarding child abuse and neglect in the Arabian Peninsula [6]. Trade associations and regional conferences provided these medical reports. This study notes that child abuse and neglect of all kinds are common in the Arab Peninsula. Most of the child abusers in this location face no consequences, are not addressed, or are even encouraged and utilized as a kind of punishment. As a result, the children who have been harmed/abused continue to suffer.

It is important to remember that children who have been mistreated or ignored may suffer significant consequences. Research indicates that 25% of abused children who are returned to their parents alone in the world experience serious re-injuries, and 5% of them die later in life. On the other hand, through thorough and intensive family treatment, 80%-90% of the families that have abused their children may be rehabilitated to give them the proper care they deserve [3].

Experts in physical and mental health generally agree that the pediatrician is the professional who is essential to the detection of abuse and/or negligence as well as the assessment of the severity of one’s abusive behavior. This is particularly true in the early years of children’s lives when pediatricians are responsible for identifying and screening young children who are at a high risk of abuse or neglect. The frequent seizures, resultant intellectual impairment, and complex treatment regimen for a patient (child or adult) with Lennox-Gastaut syndrome (LGS) require substantial effort by the parents and family [7]. 

However, owing to ethical and legal responsibilities, physicians must possess sufficient knowledge and expertise that help them identify unique physical and behavioral patterns, child abuse epidemiology, treatment, and prevention, as well as the regulations controlling their reporting duties and managing reports in their community [7]. In other words, they must be able to read between the lines and diagnose/treat cases of child abuse. In these circumstances, a successful plan requires the careful consideration of several elements [8], the first and most important of which is the professional’s awareness of his/her and his/her patient’s legal protection, along with his training and expertise regarding abuse cases. Even with such awareness and training, physicians may tend to underestimate the conditions of child abuse patients [9].

In the aforementioned context, this study was aimed at analyzing Saudi Arabian students’ indications, knowledge, views, and professional encounters regarding child abuse and/or neglect.

## Materials and methods

Study design and participants

This study was carried out in Dr. Soliman Fakeeh Hospital in Jeddah, Saudi Arabia. Its target audience comprised CAN victims as well as their relatives and kin. The study was approved by the Ethical and Research Committee in Dr. Soliman Fakeeh Hospital in Jeddah, Saudi Arabia (approval number 150IRB/2020). Informed consent was obtained from all subjects involved in the study. The study sample included a total of 65 students.

Statistical analysis

In this study, for the ratio variable as well as each interval, summary statistics were calculated. For every nominal variable, percentages and frequencies were calculated. For the ratio variables, the mean and standard deviation were calculated. Further, for several answer factors, the case percentages and replies were calculated. The scales about which the participants’ percentage scores were determined comprised physical abuse, child emotional abuse, child neglect, child sexual abuse, indications of child abuse and neglect score, COVID score, self-efficacy score, and general perception score. Cutoffs regarding good, adequate, and poor knowledge in each aforementioned domain were established using a quartile analysis of the overall knowledge score. Thus, a score >85 (upper quartile) indicated good knowledge, a score between 70 and 85 indicated acceptable knowledge and a score of 70 indicated insufficient understanding (second quartile). To ascertain whether the score variables were normal, the K-S test was implemented in this study. Moreover, the two-tailed Mann-Whitney U test was performed to observe whether there were statistically significant differences in the scores based on the levels of the research variables. Further, the effect sizes were calculated to quantify these disparities.

## Results

Summary of the baseline characteristics 

A total of 65 students participated in this study: 23 were men (35.4%) and 42 were women (64.6%). The median age (standard deviation [SD] 2.8) was 22.5 years. Among the participants, 25 individuals (23%) were in their final internship year. Notably, most of the sample consisted of lone individuals. Importantly, 61 (93.8%) and 18 (27.7%) participants were CAN victims. Concurrently, 28 participants (43%) reported that they knew someone who had passed away due to CAN. Moreover, 56 (86%) participants reported not handling any CAN cases. Further, 48 (73.8%) of them had reportedly attended a workshop or enrolled in a course on child maltreatment and neglect. The majority of students (83.1%) did not know the National Family Safety Registry (NFSR), while 78.4% of them did not know that they were supposed to dial the number 51 to report CAN cases. Throughout medical school, 24 (36.0%) individuals had reportedly undertaken one or more CAN training sessions. On the other hand, 44 individuals (67.69%) stated that they had never received any CAN instruction. Importantly, 20 (30.8%) individuals reported a rise in child abuse and neglect cases, whereas 45 (69.2%) individuals had not noticed any increase in such cases (Table 1). 

**Table 1:** Baseline characteristics of the sampled participants. The numerical data were presented as mean (standard deviation) [range], whereas the categorical data were presented as the number of cases, *n* (%).

Characteristics	Overall (*n* = 65)
Age	
Mean (standard deviation) [range]	22.5 (2.8) [17.0-33.0]
Sex, *n* (%)	
Female	42 (64.6%)
Male	23 (35.4%)
Marital status, *n* (%)	
Married	4 (6.2%)
Single	61 (93.8%)
Student or intern, *n* (%)	
Student	50 (76.9%)
Intern	15 (23.1%)
Have you ever been a victim of child abuse or negligence?	
No	47 (72.3%)
Yes	18 (27.7%)
Are you a friend or relative of a victim of child abuse or negligence?	
No	37 (56.9%)
Yes	28 (43.1%)
Have you handled incidents of child abuse and neglect in the medical industry?	
No	56 (86.2%)
Yes	9 (13.8%)
Have you attended courses/workshops/conferences/awareness campaigns regarding child abuse and neglect?	
No	48 (73.8%)
Yes	17 (26.2%)
Are you aware of the National Family Safety Registry (NFSR)?	
No	54 (83.1%)
Yes	11 (16.9%)
The number to call if you want to report a case of family violence in Saudi Arabia is…	
1919	14 (21.5%)
I do not know the number.	51 (78.4%)
Since the COVID-19 pandemic, have you been paying more attention to child abuse and neglect?	
I do not think it is important.	6 (9.2%)
I usually forget to check for signs of abuse and neglect.	9 (13.8%)
No, I only check if I am suspicious.	22 (33.8%)
No, it is just like before the pandemic.	16 (24.6%)
Yes, I have increased my attention.	12 (18.5%)
Have many cases of child abuse have you witnessed in the last four years?	
0	54 (83.1%)
1–4	11 (16.9%)
Have you noticed any increase in child abuse and neglect cases?	
No	45 (69.2%)
Yes	20 (30.8%)

Summary of the general perceptions of CAN

Out of the participant sample, 40 (61.5%) participants admitted to having received insufficient instruction regarding CAN. According to 41 (63.1%) respondents, the Saudi culture and religious beliefs needed to be considered while defining CAN. Moreover, 18 participants (27.7%) felt that the CAN supporting services currently available were adequate concerning dealing with CAN. Additionally, 38 (58.4%) of them claimed that reporting CAN incidents was preferable to dealing with them directly. Further, 40 (61.5%) participants were not aware of the CAN reporting system at their hospital, while another 17 (26.2%) of them were not sure about the same. Meanwhile, 52 individuals (82%) were willing to report suspicions about CAN. On the other hand, 16 (24.6%) participants preferred reporting a CAN case involving a major injury. Notably, 48 respondents (73.9%) thought that there weren’t enough reports of CAN cases in Saudi Arabia (Table 2).

**Table 2:** General perceptions of the study population about child abuse and neglect. The categorical data were presented as the number of cases, *n* (%).

Criteria		Strongly disagree	Disagree	Not sure	Agree	Strongly agree
GP3_ I have had adequate training to deal with child abuse and neglect.	n	18	22	21	2	2
	%	27.70	33.80	32.30	3.10	3.10
GP4_ We need to reframe child abuse and neglect in Saudi Arabia based on our culture and religion.	n	3	6	15	24	17
	%	4.60	9.20	23.10	36.90	26.20
GP5_ The current supportive services for dealing with child abuse and neglect in Saudi Arabia are competent.	n	10	15	22	10	8
	%	15.40	23.10	33.80	15.40	12.30
GP6_ I’d rather resolve a situation of child abuse and neglect on my own than report it.	n	19	19	18	6	3
	%	29.20	29.20	27.70	9.20	4.60
GP7_ I’m aware of the reporting procedure for child abuse and neglect in my hospital.	n	19	21	17	6	2
	%	29.20	32.30	26.20	9.20	3.10
GP8_ I am prepared to report any suspected incidence of child abuse and neglect.	n	0	2	11	21	31
	%	0.00	3.10	16.90	32.30	47.70
GP9_ I like to limit my reports of child abuse and neglect to those that are life-threatening.	n	20	17	12	9	7
	%	30.80	26.20	18.50	13.80	10.80
GP10_ Child abuse and neglect are underreported in Saudi Arabia.	n	1	0	16	15	33
	%	1.50	0.00	24.60	23.10	50.80

Summary of the attitudes toward CAN

In total, 51 (78.46%) participants claimed that they would report their results to the appropriate authorities, while 38 individuals (58.46%) claimed that they would share their results. Moreover, 31 respondents (47.7%) indicated they would grade the child’s and the parent’s explanation of the clinical findings. During the clinical examination, 27 (41.5%) of the participants said that they would question a kid and his/her parents regarding any observable CAN-suspicious signs or symptoms (Table 3).

**Table 3:** Attitudes toward child abuse and neglect and barriers to reporting child abuse and neglect. The categorical data were presented as the number of cases, *n* (%).

Criteria	Steps	Responses		Percentage cases
		n	%	
Attitudes toward child abuse and neglect case	Ask the kid and parents about the signs/symptoms you detect.	27	15.52	41.54
	Document the signs/symptoms and your suspicion on the file.	38	21.83	58.46
	Monitor the case during the following visits.	19	10.92	29.23
	Report to legal authorities.	51	29.31	78.46
	Consistency of parents’ and children’s explanations regarding findings.	31	17.81	47.69
	Do nothing.	1	0.57	1.54
	I do not know.	7	4.02	10.77
	Total	174	100	267.69
Barriers to reporting child abuse and neglect cases	The reporting procedure is unclear.	36	20	55.4
	Reporting child abuse or neglect to authorities is not yet acceptable in our community.	22	12.22	33.8
	There is a fear of violence or unfavorable consequences for the child	40	22.22	61.5
	There is a fear of aggressive response from the child’s family or parents.	38	21.11	58.5
	There is no legal obligation to report child abuse and neglect.	13	7.22	20
	There is uncertainty about the diagnosis of child abuse or neglect.	31	17.22	47.7
	Total	180	100	276.9

Summary of the barriers to reporting CAN

In total, 40 (61.5%) participants expressed fear regarding potential violence or negative outcomes for a child victim of CAN. Meanwhile, 38 (58.5%) of them reportedly worried about the child’s parents or other family members retaliating aggressively. Moreover, 36 participants (55.4%) did not know how to report something related to CAN. Additionally, 31 (47.7%) of them did not know that a given diagnosis was supposed to be that of CAN. On the other hand, 22 (33.8%) participants thought that their community did not yet tolerate the reporting of CAN. Also, 13 (20%) participants thought that reporting child abuse and neglect was not required by law (Table 3).

Summary of the knowledge of CAN

In this study, the five main categories utilized to assess CAN knowledge were physical abuse, emotional abuse, sexual abuse, neglect, and indicators of child abuse and neglect. In total, 50 participants (76.9%) understood physical abuse to an outstanding-to-adequate extent. Moreover, 45 (69.2%) participants reportedly possessed adequate knowledge to comprehend emotional abuse. Of those surveyed, 54 (83.1%) participants had a good enough understanding of child abuse. Meanwhile, 47 respondents (56.9%) knew enough to be well-informed about sexual abuse. In contrast, 28 (43.1%) participants had little to no knowledge of sexual abuse. Further, 50 (76.9%) of them reportedly knew enough about the CAN indication to be considered competent.

Notably, the participants’ levels of CAN knowledge did not differ in terms of gender across any categories. Moreover, the knowledge scores of participants who had experienced CAN were higher for signs of CAN (median = 84.6, *P* = 0.004), physical abuse (median = 78.3, *P* = 0.027), emotional abuse (median = 90, *P* = 0.002), and sexual abuse (median = 83.3, *P* = 0.001).

In addition, individuals with friends or family who had experienced child abuse and neglect had higher knowledge scores (median = 82, *P* = 0.043; median = 94.28, *P* = 0.00) regarding the aforementioned topics. There were no differences in the participants’ understanding of CAN across all categories irrespective of whether they had interacted with CAN cases. Regarding the tests assessing their comprehension of CAN indications, those participants who attended CAN courses or workshops achieved a better performance (median = 81.5, *P* = 0.04) (Table 4).

**Table 4:** Child abuse and neglect knowledge among students and factors affecting such knowledge. Two-tailed Mann-Whitney U test was used for comparison of median values between groups for all outcomes. Statistical significance was considered at *P *< 0.05. GP, general practitioner

n		Physical abuse score	Emotional abuse score	Neglect score	Sexual abuse score	Indicators of child abuse and neglect score
Female, *n* = 42		75.833	78	90.000	75.000	80
Male, *n* = 23		71.667	72	88.571	66.667	76.923
	U	350	413	394	413	389
	Z	–1.830	–0.962	–1.227	–0.965	–1.292
	P	0.067	0.336	0.220	0.335	0.196
	Do you have children? (Median)					
No, *n* = 61		75	76	88.571	70	80
Yes, *n* = 4		81.667	86	91.429	80	70.000
	U	97	63	92.500	95.500	93
	Z	–0.685	–1.614	–0.809	–0.727	–0.793
	P	0.494	0.107	0.418	0.467	0.428
	Have you been a victim CAN? (Median)					
No, *n* = 47		71.667	72	88.571	66.667	76.923
Yes, *n* = 18		78.333	90	92.857	83.333	84.615
	U	272.500	208.500	294.500	200.500	227.5
	Z	–2.213	–3.151	–1.893	–3.276	–2.872
	P	0.027	0.002	0.058	0.001	0.004
	Do you have a friend/relative who is a CAN victim? (Median)					
Yes, *n* = 28		80	82	94.286	80	82.308
No, *n* = 37		73.333	74	80	66.667	78.462
	U	374	365.500	237	430	398
	Z	–1.913	–2.024	–3.741	–1.171	–1.593
	P	0.056	0.043	<0.001	0.242	0.111
	Have you dealt with CAN cases? (Median)					
Yes, *n* = 9		71.667	78	85.714	80	81.538
No, *n* = 56		75	76	88.571	70	79.231
	U	243.500	236.500	231.500	201	210.500
	Z	–0.162	–0.295	–0.391	–0.973	–0.790
	P	0.871	0.768	0.696	0.331	0.430
	Have you attended CAN courses/workshops? (Median)					
No, *n* = 48		75	77	87.143	75.000	79.231
Yes, *n* = 17		78.333	78	94.286	70	81.538
	U	338	377.500	294.500	347.500	271
	Z	–1.048	–0.456	–1.703	–0.907	–2.049
	P	0.295	0.648	0.089	0.364	0.040
	Training category (Median)					
Resident/Fellow, *n* = 112		78.333	76	88.571	68.333	76.923
GP, *n* = 11		68.333	70	62.857	66.667	63.077
	U	351.500	411.500	313	563.500	481
	Z	–2.350	–1.817	–2.697	–0.468	–1.199
	P	0.019	0.069	0.007	0.640	0.231

Summary regarding CAN during the COVID-19 pandemic

Out of the sample, 34 respondents (52.3%) claimed that during the COVID-19 pandemic, cases of CAN had increased. Meanwhile, 55 (84.6%) respondents thought that physicians had to keep a closer eye out for signs of CAN during this period. Further, 53 (81.5%) respondents said that during the pandemic-induced economic lockdown, children were more likely to become victims of CAN (Table 5).

**Table 5:** Child abuse and neglect during the COVID-19 pandemic. The categorical data were presented as the number of cases, *n* (%).

Criteria		Strongly disagree	Disagree	Not sure	Agree	Strongly agree
Covid1_ Child abuse and neglect cases have increased during the COVID-19 pandemic.	n	1	3	27	13	21
	%	1.50	4.60	41.50	20.00	32.30
Covid2_ Health practitioners should be more attentive in looking for signs of child abuse and neglect.	n	0	1	9	28	27
	%	0.00	1.50	13.80	43.10	41.50
Covid3_ Children are more likely to be abused or neglected during lockdown/quarantine due to their vulnerability.	n	0	0	12	24	29
	%	0.00	0.00	18.50	36.90	44.60

## Discussion

Child abuse has become one of the most serious worldwide concerns about mental and physical well-being, given the available statistics and the therapeutic skills required to deal with child abuse cases. According to the WHO, it is a widespread issue that shows itself in many ways across the world and is strongly ingrained in cultural norms. Owing to the sensitivity and secrecy surrounding CAN, it has sparked a great deal of attention not only in Saudi Arabia but also at a global level. Some find CAN to be subjective and ambiguous owing to their diverse backgrounds, ethnicities, and faiths. Nevertheless, suspicions of child abuse must be properly documented and reported to the relevant government agencies; in turn, these agencies should investigate such cases and take appropriate measures to safeguard children from abuse [10]. If CAN diagnoses are made quickly and accurately, children who are suspected of being abused can receive the appropriate examination, investigation, and results in a streamlined way.

While CAN is a concerning issue in every country, it may go undetected and overlooked in many locations, especially in developing countries [11]. The results of studies carried out in Turkey’s eastern cities indicate that primary care physicians lack the knowledge and disposition needed to identify and report concerns regarding child abuse [12]. More recently, Saudi Arabian media have been covering child abuse cases. In this context, the present study was conducted in Saudi Arabia to evaluate doctors’ professional experiences, perceptions, and understanding of CAN, an issue that has not yet received much attention from the larger Arab community.

This study found that underreporting remains a frequent occurrence concerning CAN cases. Indeed, a significant portion of Saudi doctors concurred that although child abuse is an epidemic, it remains underreported not only in Saudi Arabia but also globally [13,14]. Furthermore, primary care doctors barely report one out of every four suspected occurrences of physical abuse of children. Although there are several facets to this issue, reluctance remains the most prevalent and pernicious one [15,16].

This study’s survey and investigation revealed that while the participants’ awareness of some key CAN concepts was sufficient, their understanding of how to report CAN incidents was comparatively inadequate. Furthermore, their professional experiences and opinions of CAN were deemed insufficient. The alarming issue of underreporting of child abuse instances in Saudi Arabia and other Arab nations could be partially reflected by these statistics.

Moreover, the results of this study showed that although participants understood numerous important CAN concepts, they lacked a robust grasp of how to report CAN events. Their professional experiences and opinions regarding CAN were also limited. The alarming problem of Saudi Arabia and other Arab countries underreporting child abuse cases could be partially reflected by such a finding.

Such a gap could result in an underestimation of the severity of the CAN problem in Saudi Arabia and, in turn, hinder the creation of measures to address it.

The importance of fostering greater confidence in healthcare facilities and professionals’ capacity to report suspected CAN cases was stressed by Borres and Hägg and Schols et al. [17,18]. These scholars suggested that by increasing knowledge, providing precise techniques, and establishing national ethical standards, CAN instances and incidence rates could be significantly reduced. However, in this respect, one must remember that CPT teams do exist in healthcare settings in Saudi Arabia, which raises questions regarding their potential impact on physicians’ attitudes and behaviors.

Notably, Saudi Arabia’s Ministry of Health mandates that healthcare professionals report any suspected cases, and all Saudi Arabian hospitals agree that CAN reporting is necessary. However, about half of the participants in the study of Li et al. believed that dentists had no moral or legal obligation to report child abuse [19]. They also found that over 80% of physicians were ignorant of their legal obligation to report suspicious cases and did not possess adequate information regarding the law enforcement agencies that needed to be contacted for the reporting of CAN cases [19].

In particular, physicians need to be well aware of the CAN reporting process and the consequences of carelessness in this respect. Even in cases when there is no proof of abuse, Saudi law guarantees complete safety for professionals and the confidentiality of reporters’ names at all times. Confidentiality and trust are also among Saudi doctors’ foremost concerns about CAN cases, according to Schols et al. and Li et al. [18,19].

Moreover, people’s decision to report CAN occurrences globally is influenced by a variety of circumstances, including a lack of skill in diagnosing the given problem, a desire to solve the situation autonomously, the risk of stigmatizing the involved family, and a lack of faith in child protection authorities. Such a decision also depends on doctors’ readiness to disclose various forms of abuse and neglect. For example, as compared to other groups, doctors remain less likely to report incidents of psychological abuse and educational neglect to the police and the welfare department. Many medical personnel admit that they fail to tell social services about child abuse, including cases when they suspect sexual assault. Instead, medical students and practitioners must maintain their will to report CAN cases after they begin clinical practice, and all physicians should get continuous education regarding the same. Notably, in the US, home-visiting programs have been instrumental in reducing child abuse. In summary, any suspicions of abuse should be immediately reported to child protective services.

In Arab nations, revealing or publicly confessing shameful or cruel behavior may be considered an undesirable breach of the concept of a *virtuous* society. Such an act can be deemed a significant violation of familial sanctity and a threat to a family’s respect and reputation, which are the most important characteristics of Arab culture. However, for the sake of one’s right to live a happy life, such a line of thinking must be disavowed.

When it comes to child abuse and neglect, a comprehensive approach to its prevention and treatment at all levels is essential to the well-being of a society and nation. Hence, the existing services and systems must include child maltreatment surveillance, preventative efforts, and care for both children and families. It is also necessary to organize a countrywide campaign in Saudi Arabia aimed at proposing rules regarding the reporting of suspected incidents of child abuse and the protection of reporting physicians. Pediatricians may play an essential role in guiding such procedures. To this end, Saudi Arabia requires the collaborative and synchronized forces of several of its industries. It must ensure that medical students/practitioners in the nation can detect the symptoms and warning signs of child abuse and neglect. Such professionals must provide practical learning opportunities in the domains of family medicine or pediatrics so that their students may learn how skilled medical professionals handle such situations.

The small sample size was a limitation of the study.

## Conclusions

This study claims that a comprehensive plan aimed at the prevention and treatment of child maltreatment at all levels is essential in Saudi Arabia, especially given the current state of professional knowledge and clinical experience on the issue of child abuse and neglect in this nation. Hence, the existing services and systems must include child maltreatment surveillance, preventative efforts, and care for both children and families. It is also necessary to organize a countrywide campaign in Saudi Arabia aimed at proposing rules regarding the reporting of suspected incidents of child abuse and the protection of reporting physicians. Pediatricians may play an essential role in guiding such procedures. It must ensure that medical students/practitioners in the nation can detect the symptoms and warning signs of child abuse and neglect as well as be aware of the ethical and legal responsibilities for reporting the same. Such professionals must provide hands-on learning opportunities in the domains of family medicine or pediatrics so that their students may learn how competent medical professionals handle situations of child abuse and neglect. In the instances of CAN, they must encourage the affected children to actively engage while keeping an eye on them.
